# Effect of different concentrations of omega-3 fatty acids on stimulated THP-1 macrophages

**DOI:** 10.1186/s12263-017-0554-6

**Published:** 2017-02-21

**Authors:** B. Allam-Ndoul, F. Guénard, O. Barbier, M-C Vohl

**Affiliations:** 1 Institute of Nutrition and Functional Foods (INAF), Laval University, Pavillon des Services, 2440 Hochelaga Blvd, Québec, Québec Canada; 2 Laboratory of Molecular Pharmacology, CHU de Québec Research Center, 2705 Laurier Blvd, Québec, Québec G1V 4G2 Canada

**Keywords:** EPA, DHA, LPS, Inflammation, mRNA

## Abstract

**Background:**

Inflammation plays a central role in chronic diseases occurring in the contemporary society. The health benefits of omega-3 (n-3) fatty acids (FAs), mostly eicosapentaenoic acid (EPA) and docosahexaenoic acid (DHA), have been reported. However, their mechanisms of action are poorly understood. We explored dose and time effects of EPA, DHA, and a mixture of EPA + DHA on the expression of inflammatory genes in stimulated macrophages.

**Methods:**

Lipopolysaccharide was used to stimulate human THP-1 macrophages. Cells were incubated in different conditions in the presence of n-3 FAs and LPS, and mRNA levels of inflammatory genes were measured by real-time PCR. Cytokine levels in culture media were measured.

**Results:**

The mixture of EPA + DHA had a more effective inhibitory effect than either DHA or EPA alone, DHA being more potent than EPA. For both EPA and DHA, 75 μM of FAs had a more important anti-inflammatory effect than 10 or 50 μM. For gene expression, EPA had the greater action during the post-incubation (after LPS treatment) condition while DHA and EPA + DHA were more potent during the co-incubation (n-3 FAs and LPS). Cytokine concentrations decreased more markedly in the co-incubation condition.

**Conclusions:**

These results suggest that in stimulated macrophages, expression levels of genes involved in inflammation are influenced by the dose, the type of n-3 FAs, and the time of incubation.

## Background

Inflammation is a biological response to harmful chemical or physical stimuli. The purpose of an acute resolving inflammatory response is to protect the human body from damage and to re-establish homeostasis [24]. Several conditions such as inflammatory bowel disease, cardiovascular diseases (CVD), Alzheimer’s disease, or cancers are caused by a persistent inflammation and their prevalence increase due to inappropriate responses arising in a chronic manner along with them [9, 20].

Fatty acids (FAs) are carboxylic acids with a variable number of carbon atoms, building a hydrocarbon chain, which finishes with carboxyl and methyl groups. Polyunsaturated FAs (PUFAs) contain more than one double bond in their backbone. Omega-6 (n-6) and omega-3 (n-3) are the two main families of FAs. Linoleic acid (LA) and alpha-linolenic acid (ALA) are, respectively, the precursors of n-6 and n-3 FAs. These FAs are called essential because they cannot be produced by the body and must be provided by the diet. The main FAs derived from n-6 FAs is arachidonic acid producing rather pro-inflammatory eicosanoids. FAs derived from ALA, namely eicosapentaenoic acid (EPA; 20:5n − 3) and docosahexaenoic acid (DHA; 22:6n − 3), produce eicosanoids with a less inflammatory profile [4]. Human being synthetizes EPA and DHA from their dietary precursor ALA at a very low rate. This makes dietary intake of EPA and DHA a more efficient source for their assimilation. In fact, their availability are increased when they are found in the flesh of oily fish such as herring, mackerel, and salmon or in fish oil supplements [19].

n-3 FAs, particularly EPA and DHA, have been proven to exert beneficial effects on health. Over the past decades, mechanisms by which EPA and DHA impact inflammation have been investigated, demonstrating an improvement of cardiovascular health [10, 14], inflammation status [5], or cancer progression [3, 12] after their consumption.

Despite significant advances on health benefits associated with n-3 FAs, the dose needed to provide health benefits, the best window of time to consume them to fight efficiently against inflammation, and the mechanisms of action in preventing inflammation-related diseases are still unclear. Thus, the aim of the present study was to investigate the effect of 10, 50, and 75 μM EPA, DHA, and a mixture of EPA + DHA on the expression of inflammatory genes in stimulated THP-1 macrophages. The impact of the condition of incubation (pre-incubation, co-incubation, post-incubation) with n-3 FAs relative to macrophage stimulation was also evaluated.

## Methods

### Reagents and cell lines

Cell culture media, Roswell Park Memorial Institute (RPMI) 1640 medium, fetal bovine serum (FBS), penicillin and streptomycin media supplements, phorbol 12-myristate 13-acetate (PMA), and dimethyl sulfoxide (DMSO) were purchased from Thermo Scientific (Walthman, USA). Lipopolysaccharide (LPS) from *Escherichia coli* 0111:B4 (reference L2630) was purchased from Sigma (Saint Louis, USA). Phosphate-buffered saline (PBS) solution was obtained from Life Technologies (Burlington, Canada). EPA, DHA, and reagents for reverse transcription were obtained from Applied Biosystems (Oakville, Canada).

### Cell culture and fatty acid treatment

The human THP-1 cell line, an acute monocytic leukemia cell line (American Type Culture Collection (ATCC), Rockville, MD, USA), was cultured in RPMI 1640 media supplemented with penicillin (100 U/ml) and streptomycin (100 μM/ml), 10% FBS at 37 °C in a 5% CO_2_ incubator. Differentiation of monocytes into macrophages was induced with PMA. 9 × 10^5^ cells per ml were seeded into six-well plates, with 200 nM of PMA for 72 h. Then, non-attached cells were removed by aspiration, adherent cells were washed three times with PBS, and then, cells were ready for experiments. The cells were incubated in different conditions: (1) in the post-incubation condition, the macrophages were stimulated during 18 h by LPS, before the addition of n-3 FAs for 24 h; (2) in the co-incubation condition, the cells were incubated during 24 h with LPS and n-3 FAs at the same time; (3) finally, in the pre-incubation condition, the macrophages were incubated during 24 h into n-3 FAs and then stimulated during 18 h by an addition of LPS.

### n-3 FAs and LPS preparation

All treatments were performed in triplicate, and the entire experiment was replicated independently three times. LPS was dissolved in PBS and diluted to a final concentration of 10 ng/ml prior to treatment. Stock solutions of FAs (EPA-DMSO 33 × 10^4^ μM and DHA-DMSO 76 × 10^4^ μM) prepared in serum-free RPMI 1640 medium were diluted in culture medium to obtain 10, 50, and 75 μM concentrations. Fresh FAs and LPS were prepared before every experiment from the frozen stock solution. The cells were thereafter incubated with LPS and EPA, DHA, or EPA + DHA (ratio 1:1) for 24 h. Controls in this experiment were THP-1 cells incubated with the vehicle, DMSO, and LPS.

### Cell proliferation and cytotoxicity assay

A viability test was performed to exclude cytotoxicity of EPA, DHA, and EPA + DHA concentrations used. Briefly, cell cytotoxicity was assessed by measuring the activity of mitochondrial dehydrogenase. 3-(4,5-Dimethyl-2-thiazol)-2,5-diphenyl-2h-tetrazolium bromide (MTT) reagent was used. After incubation at 37 °C for 1 h, the absorbance at 490 nm was assayed using an ELISA plate reader (Biotech).

### RNA isolation and quantitative real-time PCR

After 24 h, following the protocol provided, total RNA was extracted using RNeasy Mini Kit (Qiagen). RNA quality and integrity were tested on 1.5% agarose gel electrophoresis with ethidium bromide staining. Absorption spectroscopy at 260/280 was used to determine RNA concentrations. Then, complementary DNA (cDNA) was produced from RNA using High Capacity Transcription Kit (Applied Biosystems). The expression of several inflammatory genes (*SOCS1*, *TNFAIP3*, *TNFA*, *IL1B*, *IL6*, *MCP1*, *PTGS2*) was assessed using real-time PCR. PCR samples were normalized against 18S gene expression. Applied Biosystems provided primers and TaqMan® probes (Table 1).

**Table 1:** Probe sets for real-time PCR

	5′ Forward primer
	3′ Reverse primer
SOCS1	TCCTGAGGAGCGGGAGGAGTGGAC
	GTCCACTCCTCCCGCTCCTCAGGA
TNFAIP3	GAAAACGACGGTGACGGCATTGC
	GCAATGCCGTCCGTCGTCGTTTTC
TNFA	CCATGTTGTAGCAAACCCTCAAGCT
	AGCTTGAGGGTTTGCTACAACATGG
IL1B	CAGATGAAGTGCTCCTTCCAGGACC
	GGTCCTGGAAGGAGCACTTCATCTG
MCP1	CGCTCAGCCAGATGCAATCAATGCC
	GGCATTGATTGCATCTGGCTGAGCG
PTGS2	CTGGGCCATGGGGTGGACTTAAATC
	GATTTAAGTCCACCCCATGGCCCAG
IL6	TCAGCCCTGAGAAAGGAGACATGTA
	TACATGTCTCCTTTCTCAGGGCGA
18S	CCATTGGAGGGCAAGTCTGGTGCCA
	TGGCACCAGACTTGCCCTCCAATGG

### Cytokine measurements

Cytokines, TNFA, IL1B, IL6, and MCP1, were assessed in collected media, using the Bio-Plex Pro Human Chemokine kit (Bio-Rad Laboratories Canada Ltd., Mississauga, ON, Canada) according to the manufacturer’s instructions.

### Statistical analyses

Statistical analyses were done using SAS software (version 9.2). Experimental results were reported as mean ± SE. Analysis of variance was used to compare between group means. A significant overall *F* test was followed by post hoc comparisons using the LS means procedure. The level of significance was defined at *P* < 0.05 for all.

## Results

### Cytotoxicity assay

The effect of EPA, DHA, and EPA + DHA on THP-1 macrophage viability was performed using the MTT assay. After 24-h incubation of the cells with 10, 50, and 75 μM EPA, DHA, and EPA + DHA, the test was done. All concentrations of each n-3 FA investigated did not have any effect on cell viability (data not shown).

### Influence of EPA, DHA, and EPA + DHA on inflammatory gene expression

**Fig. 1:**
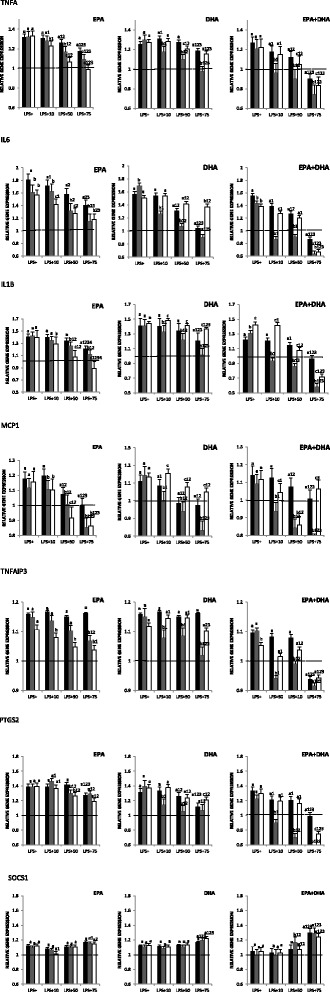
Influence of n-3 FAs on gene expression. The total RNA was extracted using RNeasy Mini, then cDNA was produced from the RNA. The expression of *SOCS1*, *TNFAIP3*, *TNFA*, *IL1B*, I*L6*, *MCP1*, and *PTGS2* was assessed using real-time PCR. PCR samples were normalized against 18S gene expression. Gene expression was measured under three conditions. ^*a*, *b*, *c*^Represents the differences (*P* ≤ 0.05) between each of these conditions (pre-incubation [*black bars*], co-incubation [*gray bars*], and post-incubation [*white bars*]). ^1^ *P* ≤ 0.05 represents the difference between different concentrations relative to LPS+ within the same condition; ^2^ *P* ≤ 0.05 represents the difference between different concentrations relative to 10 μM within the same condition; and ^3^ *P* ≤ 0.05 represents the difference between different concentrations relative to 50 μM within the same condition

The cells were cultivated in three conditions. First, the macrophages were incubated in FAs before being stimulated (pre-incubation). In the second condition, inflammation and treatment with n-3 FAs were done at the same time (co-incubation). Finally, in the last condition, inflammation was triggered before adding the FAs (post-incubation). In the presence of LPS stimulation, there was a global increase of gene expression levels of inflammatory genes compared with the control (dotted line, Fig. 1). After the addition of n-3 FAs in the culture medium, a global suppression of *IL6*, *TNFA*, *IL1B*, *MCP1*, *TNFAIP3*, and *PTGS2* expression was seen. The effect was more pronounced with the mixture EPA + DHA than with either EPA or DHA alone. The incubation of cells with n-3 FAs had different effects depending on the FAs. Except for *MCP1* for which the post-incubation and co-incubation conditions had the same effect, the post-incubation with EPA was more efficient on reducing *IL6*, *TNFA*, *IL1B*, and *TNFAIP3* gene expression than the co-incubation and the pre-incubation. For treatment with either DHA or the mixture EPA + DHA, the co-incubation of THP-1 cells in these FAs was more potent than the pre- and post-incubation for all the studied genes. A dose effect was observed on gene expression levels; incubation of cells in 75 μM of any FAs decreased their expression more efficiently than incubation in 10 and 50 μM. LPS stimulation increased *SOCS1* expression. A regulation of this gene was seen only with 75 μM of each n-3 FA, which further increased *SOCS1* expression. No condition-dependant modulation of *SOCS1* was seen.

### Influence of EPA, DHA, and EPA + DHA on cytokine secretion

Since the results obtained in the co-incubation and the post-incubation conditions were the most interesting, cytokine measurements were done for these two conditions.

**Fig. 2:**
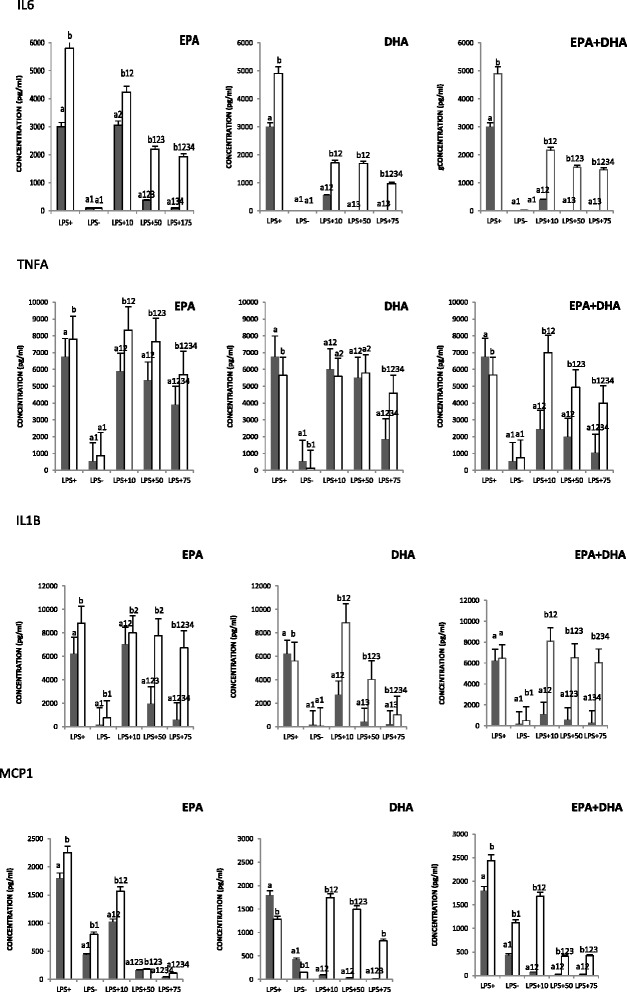
Influence of n-3 FAs on cytokine secretion. TNFA, IL1B, IL6, and MCP1 were assessed in collected media of cell cultures. ^a, b^Represents the differences (*P* ≤ 0.05) between each condition (co-incubation [*gray bars*] and post-incubation [*white bars*]). ^1^ *P* ≤ 0.05 represents the difference between different concentrations relative to LPS+ within the same condition; ^2^ *P* ≤ 0.05 represents the difference between different concentrations relative to LPS− within the same condition; ^3^ *P* ≤ 0.05 represents the difference between different concentrations relative to 10 μM within the same condition; and ^4^ *P* ≤ 0.05 represents the difference between different concentrations relative to 50 μM within the same condition

In the LPS-stimulated condition, compared to the control condition (LPS−), there was an increased production of cytokines (Fig. 2). When EPA, DHA, or the mixture EPA + DHA was added to the cultured medium, for each cytokine, a decreased secretion was observed. This diminution was more important in the co-incubation than in the post-incubation condition. A dose effect was also noted; 75 μM of each n-3 FA decreased cytokine concentrations more effectively than 10- and 50-μM doses. FA-specific effects were also seen. DHA and EPA + DHA downregulated cytokine secretion more efficiently than EPA. For IL1B and IL6, the co-incubation with DHA and EPA + DHA restored secretion back to the basal level. This phenomenon was not observed in cells incubated with EPA.

## Discussion

Because of the health benefits associated with DHA and EPA, several international agencies and organizations have put together recommendations for EPA and DHA supplementation and for fish consumption [10]. This initiative enabled not only health promotion but also the reduction of the risk of several chronic inflammatory diseases [3, 23]. Although much has been learn about n-3 FAs, many questions remain, including the dose-response effect on clinical outcomes as well as the differential effects on health of EPA and DHA.

The objective of the present study was to investigate the effect of different concentrations of EPA, DHA, and EPA + DHA on inflammatory markers, in stimulated THP-1 macrophages. It is well accepted that EPA and DHA are associated with a lower risk of inflammatory diseases [7]. Their health effects are usually studied as a sum or combination. Thus, little is known about the potential health effect of EPA compared to DHA. Nevertheless, few studies have shown EPA- or DHA-specific effects on inflammation. Gorjao and collaborators reported different effects of EPA and DHA on endothelial cells, insulin-secreting cells, and leukocyte function [8]. In a study on RAW 264.7 macrophages, Honda et al. demonstrated that EPA and DHA attenuated cell inflammatory activities and more importantly that their respective effect changed in potency depending on the investigated cytokines (IL6 and TNFA) [11]. Finally, an investigation of the effect of EPA and DHA on THP-1 macrophages showed that EPA and DHA had a differential effect on cytokine transcription [17]. It is important to further understand the specific effect of n-3 FAs on inflammatory mechanisms and to know whether they have complementary, shared, or divergent effects. A previous study by our research group [2] investigated the effect of 50 and 10 μM EPA, DHA, and EPA + DHA on non-stimulated macrophages. This study firstly showed that in non-inflammatory conditions, EPA and DHA had a varying effect on the expression of inflammatory genes and that the anti-inflammatory effect was dose-dependent [2].

In the present study, our ultimate goal was to investigate the anti-inflammatory effects of n-3 FAs in inflamed cells. In this perspective, the current study investigated the effect of EPA, DHA, and EPA + DHA on stimulated THP-1 macrophages. n-3 FA effects were tested on seven genes involved in inflammation. An incubation of these macrophages into 100 ng/ml LPS for 18 h allowed mimicking an inflammatory state. Stimulation with LPS increased expression levels of inflammatory genes.

The combination of EPA + DHA (1:1) had a more potent anti-inflammatory action than DHA and EPA alone, DHA being a better inhibitor of the studied gene expression than EPA. This implies that giving a mixture of EPA + DHA (1:1) seems to have the best anti-inflammatory effect. The dose of 75 μM was more powerful than 10 and 50 μM. A treatment of T cells with 12.5 μM EPA or DHA during 24 h was done by Verlengia and collaborators [22]. Using the microarray technique, the expression of specific selected genes involved in cytokine production, cell production, signal transduction, and apoptosis was changed. DHA increased the expression of 62% of the studied genes against 33% for EPA. In Raji cells which were treated in the same conditions, 25.9% of the studied genes were regulated by EPA against 8.4% by DHA. Only 3% of the genes were regulated by the two n-3 FAs [22]. These results suggest that molecular mechanisms responsible for the modulatory effect of EPA and DHA on T lymphocytes are different and that DHA regulated a bigger proportion of studied genes. In line with different modulatory effects of EPA and DHA revealed by us and others, an effect of n-3 FAs on heart rate mediated by DHA rather than EPA was previously demonstrated in a supplementation study among humans [15]. High heart rate (HR) has long been associated with CVD morbidity in epidemiological studies. It has been proven that fish oil intake reduced HR mostly in individuals with a high baseline HR and when taken for a long intervention period. In fact, DHA alone (2.8 g/day) diminished HR by 7% in post-menopausal women and DHA but not EPA reduced HR by 3.5 beats per minute and 2.2 beats per minute, respectively, in hyperlipidemic and healthy males [15].

Regarding cytokine production, it was mostly inhibited with the mixture of EPA + DHA. This inhibition was more pronounced than the one observed with either DHA or EPA alone, DHA being more efficient than EPA. Weldon et al. investigated the action of n-3 FAs on cytokine expression in THP-1 macrophages [17]. Pre-treatment with 100 μM EPA and DHA decreased LPS-stimulated THP-1 cell secretion of TNFA, IL1B, and IL6 compared to control. Even though the effect of the mixture of EPA + DHA was not investigated, the effect of DHA was more important than the one observed with EPA. Similar results were obtained when a lower dose was used, 25 μM DHA decreased the production of IL6 and IL1B more potently than EPA in LPS-stimulated THP-1 macrophages. A similar observation has been reported after addition of EPA and DHA to the culture media of RAW 264.7 macrophages [11]. It must also be noted that in the present study, n-3 FAs decreased IL6 secretion to a greater extent than other cytokines. This corroborates the fact that anti-inflammatory effects of n-3 FAs were cytokine specific rather than global.

To our knowledge, only one study has been designed to provide a head-to-head comparison of the effect of EPA and DHA on inflammation markers as a primary outcome. Allaire and collaborators provided 2.7 g/day of EPA and DHA for 10 weeks to healthy people with abdominal obesity. The group which had DHA supplement had a greater reduction of plasma IL8 levels and greater increase of adiponectin concentration [1].

The present study showed that in inflammatory conditions, DHA has a better anti-inflammatory effect than EPA. It also showed that the mixture EPA/DHA at a 1:1 ratio more efficiently inhibits inflammation. In human subjects, n-3 FAs have been shown to modulate inflammation-related conditions such as hypertension, dyslipidemia, or insulin resistance [4]. However, it should be noted that, up to now, most trials have not considered that the relative proportion of EPA and DHA may influence the results. This phenomenon might, at least in part, explain the relative controversy on the beneficial effect of n-3 FAs on several conditions. Luis and collaborators [13] have investigated on Wistar Kyoto rats the effect of a supplementation with different ratios of EPA + DHA on markers of CVD and oxidative stress. EPA + DHA at a 1:1 ratio triggered the most important improvement of the risk factor for type 2 diabetes and reduction of oxidative stress. It is important to point out the fact that in most studies, supplements used have a greater proportion of EPA than DHA. Since DHA appears to have a more important anti-inflammatory action, it might be interesting to reconsider their ratio in supplements, at least for inflammatory diseases.

At the present time, there is no consensus on the ideal n-3 FA intake. Nutritional guidelines have been set by several governments (France, Belgium, Canada) and health organizations (Food and Agriculture Organization, American Dietetic Association) [21]. For instance, the American Heart Association (AHA) recommends that all adults eat fish at least twice a week, which provides 500 mg n-3 FAs/day. It is also recommended for people with documented coronary heart diseases to consume 1 g EPA and DHA/day for secondary prevention [6]. Mozaffarian and collaborators recently reviewed facts concerning EPA and DHA possible shared or complementary effects [16]. In human and animal studies, EPA and DHA reduced platelet aggregation, modulate inflammation, and lower plasma triglyceride (TG) levels. Clinical and observational studies show that DHA increases high-density lipoprotein particles, favors the proportion of large low-density lipoprotein, and causes stronger TG-lowering effects. Mozaffarian et al. concluded that EPA and DHA have complementary and shared benefits. In a study led by Robinson et al., 3T3-L1 adipocytes were incubated in 125 μM of several fatty acids among which are EPA and DHA. Results have shown that DHA increased cellular adiponectin mRNA and secreted adiponectin protein to a greater extent (40% more, *P* < 0.05) than EPA [18].

Unfortunately, scientific evidences are still lacking to make quantitative recommendation about the ratio and the dose of EPA and DHA that should be taken to prevent inflammatory diseases. However, it is known that n-3 FAs have anti-inflammatory effects and thus their consumption should be favored. Additional experimental, clinical, and observational investigations are necessary to better understand the complementary and shared effects of EPA and DHA on various clinical outcomes.

A dose effect was obvious for the studied genes; 75 μM of each FA had a stronger anti-inflammatory effect than 10 and 50 μM. It must be noted that a decrease of the expression of all genes except for *SOCS1* was seen after the addition of 75 μM n-3 FAs. *SOCS1* gene expression was increased. This gene encodes for a suppressor of cytokine signaling. This result suggests that 75 μM but not 10 or 50 μM of EPA, DHA, or EPA + DHA was able to increase *SOCS1* expression. *SOCS1* being an anti-inflammatory gene, here again, a protective effect of n-3 FAs is showed.

We tested the effect of three different concentrations of each n-3 FA in three different cell culture conditions. In the first condition, the macrophages were incubated in FAs before being stimulated (pre-incubation); in the second, the inflammation and the treatment with n-3 FAs were done at the same time (co-incubation); and in the last condition, inflammation was triggered before adding the FAs (post-incubation). In the post-incubation condition, the anti-inflammatory effect of EPA was greater for *IL6*, *IL1B*, *TNFA*, and *TNFAIP3*. As far as *MCP1* was concerned, the post- and co-incubation had the same effect. EPA + DHA had the best anti-inflammatory effect during the co-incubation for all genes except for *SOCS1* where no differences were observed between each condition. Concerning cytokine secretion, the co-incubation seems to be the better condition for EPA, DHA, and EPA + DHA. Globally, findings from gene expression and cytokine production suggest that according to the inflammatory environment, the action of each n-3 FA could be different. Thus, EPA seems to have a greater anti-inflammatory effect on a situation where an inflammatory state is already present; this can be associated to a resolution of inflammation. DHA and the mixture EPA + DHA have a more potent action when they were added at the same time with LPS. This suggests that starting to take EPA and DHA at the same moment with the installation of inflammation might bring out their best anti-inflammatory effects. Even if the anti-inflammatory effect of n-3 FAs on the pre-incubation condition is the less important, a dose effect does exist. It suggests that the protective effect of n-3 FAs among healthy subjects might be visible but less obvious than the one observed among people with pre-existing inflammatory conditions. Current recommendations concerning the consumption of n-3 FAs are available not only as primary prevention of healthy people but also as secondary prevention for people suffering of coronary heart disease or having high TG levels. Human studies investigating the impact of n-3 FA intake against non-CVD were unfortunately not consistent with the data collected in pre-clinical studies. This is why consensus recommendation has not yet been made regarding the possible curative effects of these FAs or their ability to prevent inflammatory disorders. Further investigations are needed to build more precise recommendations which would take into account the body inflammatory state, the best n-3 FAs, and the dose to provide in different inflammatory states.

## Conclusions

The n-3 FAs do not have the same anti-inflammatory effect, and depending on the inflammatory state, their action can be different. This observation adds to the complexity concerning the dose and ratio of n-3 FAs for optimal health benefits. In fact, their action seems to depend on their respective dose when used alone, the ratio of each of them when used in combination, and also the inflammatory environment at the time of their consumption.

## Abbreviations

ALA: Alpha-linolenic acid
DHA: Docosahexaenoic acid
DMSO: Dimethyl sulfoxide
EPA: Eicosapentaenoic acid
FAs: Fatty acids
FBS: Fetal bovine serum
LA: Linoleic acid
LPS: Lipopolysaccharide
n-3: Omega-3
n-6: Omega-6
PMA: Phorbol 12-myristate 13-acetate
PUFAs: Polyunsaturated FAs
RNA: Ribonucleic acid
RPMI: Roswell Park Memorial Institute
